# Intratumoral and intracranial hemorrhage associated with MAPK-pathway targeted therapy: a systematic review and mechanistic synthesis

**DOI:** 10.1007/s11060-026-05714-0

**Published:** 2026-07-15

**Authors:** Sudarshawn Damodharan, Alejandra Calderon, Mohamed S. Abdelbaki

**Affiliations:** 1 Division of Pediatric Hematology, Oncology and Bone Marrow Transplant, University of Chicago, 5841 S. Maryland Avenue, Chicago, IL 60637 USA; 2 Division of Pediatric Hematology and Oncology, Fundación Santa Fe de Bogotá, Universidad de los Andes, Bogotá, Colombia; 3 Division of Pediatric Hematology, Oncology and Bone Marrow Transplant, St. Louis Children’s Hospital, Washington University School of Medicine, St. Louis, MO USA

**Keywords:** MAPK pathway inhibitor, Intratumoral hemorrhage, Intracranial hemorrhage, Pediatric low-grade glioma, Melanoma brain metastases

## Abstract

**Purpose:**

MAPK-pathway inhibitors including BRAF, MEK, and type II RAF inhibitors are now integral to treatment of pediatric low-grade glioma (pLGG), BRAF V600–mutant glioma, NF1-associated tumors, and melanoma brain metastases. Intratumoral and intracranial hemorrhage has emerged as a clinically relevant safety signal, but drug-specific incidence estimates, phenotypic definitions, and contributing mechanisms remain incompletely characterized. We systematically reviewed the evidence and synthesized contributing mechanisms.

**Methods:**

We performed a systematic review of PubMed/MEDLINE and Embase from database inception through May 2026, supplemented by FDA prescribing information, to identify studies reporting intratumoral or intracranial hemorrhage with MAPK-pathway targeted agents across tumor types. Results were synthesized narratively given heterogeneity in reporting definitions and study designs.

**Results:**

CNS hemorrhage signals were identified across agents. Dabrafenib-based regimens in melanoma brain metastases produced ICH rates of 6% (BREAK-MB monotherapy) and a fatal hemorrhage in 0.8% (COMBI-MB combination). In the phase 2 FIREFLY-1 trial of tovorafenib in BRAF-altered pLGG (*n* = 137), any-site hemorrhage of any grade occurred in 42% of patients with grade 3–4 hemorrhage in 7 patients (5%) and, separately, one fatal grade 5 intratumoral hemorrhage; in the overlapping pooled FDA-label safety population (*N* = 140), intratumoral hemorrhage specifically was reported in 9%. Combining BRAF inhibitors with stereotactic radiosurgery was associated with 3-fold higher odds of ICH versus radiosurgery alone (OR 3.16; 95% CI 1.43–6.96).

**Conclusion:**

MAPK-pathway inhibitors are associated with a consistent but heterogeneous CNS hemorrhage signal. Reported incidence varies by agent, population, and hemorrhage ascertainment method (active imaging-based surveillance versus clinical reporting), from < 1% symptomatic ICH with dabrafenib in adult melanoma to 9% intratumoral hemorrhage in the pooled pediatric tovorafenib safety population. Standardized definitions, CNS-specific CTCAE capture, and prospective imaging surveillance are needed to define true incidence and risk as MAPK-directed therapy expands in neuro-oncology.

**Registration:**

This systematic review was not prospectively registered in PROSPERO.

**Supplementary Information:**

The online version contains supplementary material available at 10.1007/s11060-026-05714-0.

## Introduction

Constitutive activation of the mitogen-activated protein kinase (MAPK) pathway is a defining oncogenic driver in many pediatric and adult central nervous system (CNS) tumors. BRAF fusions, BRAF V600E mutations, and NF1 loss converge on RAS–RAF–MEK–ERK signaling and together account for the majority of molecular alterations in pediatric low-grade glioma (pLGG), the most common childhood brain tumor [1, 2]. Targeted inhibition of this pathway with BRAF inhibitors, MEK inhibitors, BRAF/MEK combinations, and, more recently, type II RAF inhibitors has redefined treatment of pLGG [3–6], pediatric BRAF V600–mutant high-grade glioma [7], neurofibromatosis type 1 (NF1)–associated plexiform neurofibromas [8], and BRAF V600–mutant melanoma with brain metastases [9, 10].

Tovorafenib (Ojemda), a once-weekly oral, CNS-penetrant type II RAF inhibitor with activity against both BRAF fusions/rearrangements and BRAF V600 alterations, received accelerated FDA approval in April 2024 for relapsed or refractory BRAF-altered pLGG on the basis of the phase 2 FIREFLY-1 trial [3, 11]. The initial FIREFLY-1 report described an overall response rate (ORR) of 67% by the prespecified RANO-HGG criteria, with a median duration of response of 16.6 months [3]; the FDA approval summary, using the more disease-appropriate RAPNO-LGG criteria in the registrational population (*n* = 76), reported an ORR of 51% and a median duration of response of 13.8 months [11, 12]. Together these results underscore the therapeutic value of sustained MAPK-pathway inhibition in BRAF-driven disease, but they also emphasize the importance of characterizing the toxicity profile as these agents move into earlier lines of therapy and broader CNS populations.

As MAPK-directed therapy moves into earlier lines and more CNS tumor populations, rare but serious toxicities including intratumoral and intracranial hemorrhage have become a recognized concern [13]. Intratumoral hemorrhage is already an established complication of selected primary and metastatic brain tumors, particularly melanoma [14], but a distinct signal has emerged with MAPK-pathway inhibition itself: hemorrhage is a labeled warning for selumetinib [15] and tovorafenib [12], and fatal CNS bleeding has been reported with dabrafenib-based regimens in melanoma brain metastases [9, 10]. These observations are scattered across tumor types, agents, and reporting frameworks, with inconsistent phenotyping of CNS versus non-CNS bleeding, variable use of radiographic surveillance, and limited integration with co-exposure data. Together, these gaps preclude unified incidence estimates or evidence-based monitoring guidance.

We therefore performed a systematic review to synthesize published incidence estimates of intratumoral and intracranial hemorrhage associated with MAPK-pathway targeted therapy across tumor types, characterize agent-level differences in hemorrhage phenotype and severity, and propose a mechanistic and clinical framework to inform monitoring and future trial design in neuro-oncology.

## Methods

### Search strategy and data sources

A systematic search of PubMed/MEDLINE and Embase was conducted from database inception through May 29, 2026, following Preferred Reporting Items for Systematic Reviews and Meta-Analyses (PRISMA) principles [16]. Search terms combined hemorrhage descriptors (“intratumoral hemorrhage,” “intracranial hemorrhage,” “tumor hemorrhage,” “bleeding”) with MAPK-pathway agents and drug classes (“MEK inhibitor,” “BRAF inhibitor,” “RAF inhibitor,” “selumetinib,” “mirdametinib,” “trametinib,” “cobimetinib,” “binimetinib,” “dabrafenib,” “vemurafenib,” “encorafenib,” “tovorafenib”). The search was supplemented by review of FDA approval packages and prescribing information, and by hand-searching reference lists of included articles and relevant narrative reviews.

### Eligibility criteria

We included human studies that reported intratumoral or intracranial hemorrhage temporally associated with exposure to a MAPK-pathway targeted agent (BRAF inhibitor, MEK inhibitor, BRAF/MEK combination, or type II RAF inhibitor), in any tumor type. The review centered specifically on intratumoral (ITH) and intracranial (ICH) hemorrhage; non-CNS bleeding events (e.g., epistaxis) were recorded only where the source reported them together with CNS events, to provide context rather than to be interpreted as CNS hemorrhage. Studies of BRAF-mutant melanoma were included when they reported outcomes in patients with brain metastases; because some melanoma safety datasets pool patients with and without documented intracranial disease, denominators drawn from such datasets may not correspond exclusively to patients at risk for CNS hemorrhage and are interpreted with that limitation in mind. Eligible designs included prospective and retrospective clinical trials, regulatory safety summaries, observational cohorts, case series, and case reports. Narrative reviews without original patient-level or trial-level data were excluded from data extraction but screened for citation chaining. Non-human studies, preclinical reports, conference abstracts without sufficient denominator information, and non-English-language publications without available translation were excluded.

### Data extraction and synthesis

**Fig. 1:**
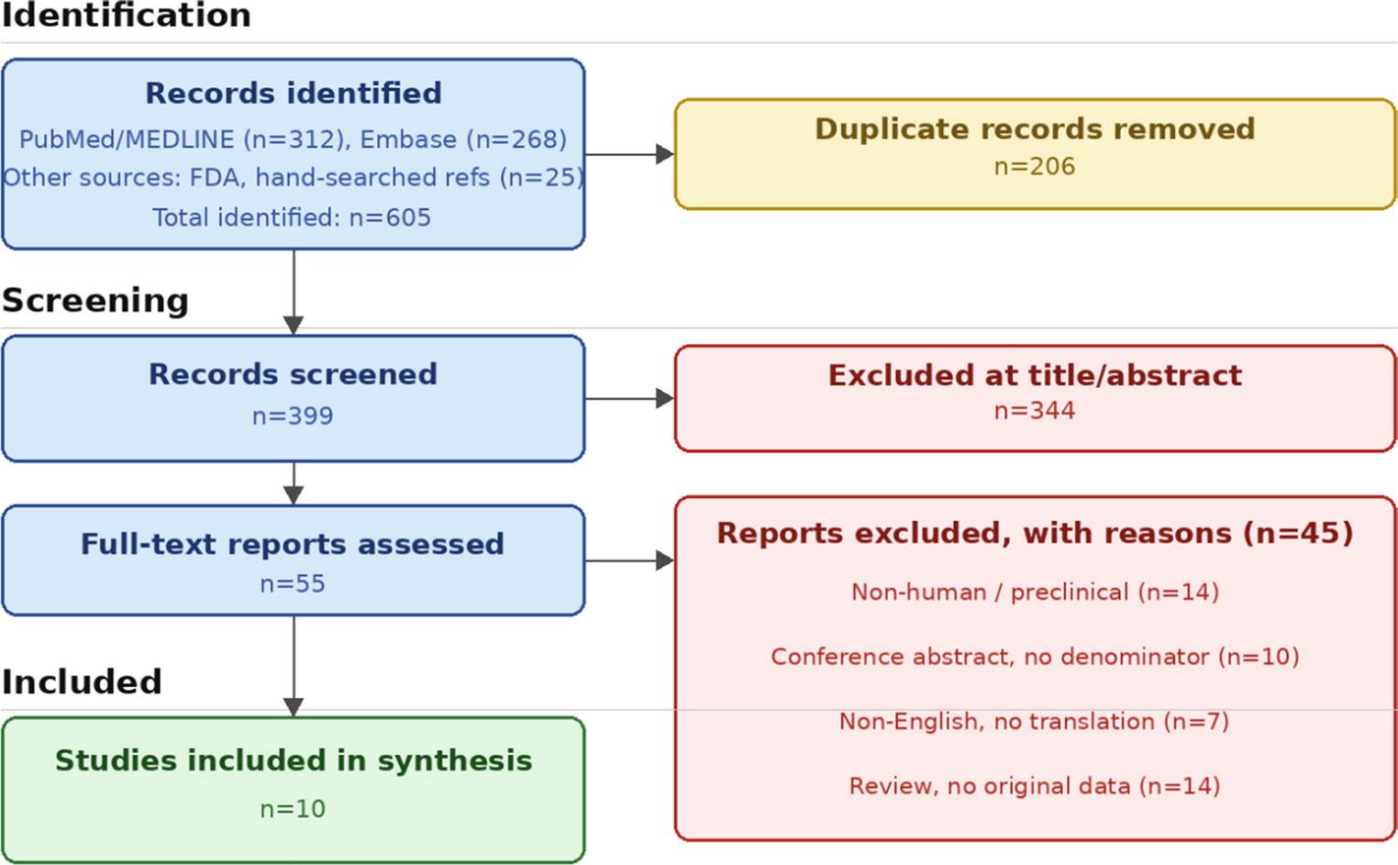
PRISMA 2020 flow diagram of study selection. A systematic search of PubMed/MEDLINE (*n* = 312) and Embase (*n* = 268) from database inception through May 29, 2026 identified 580 records, supplemented by 25 additional records obtained from US Food and Drug Administration approval packages, prescribing information, and hand-searching of reference lists. After removal of 206 duplicates, 399 records underwent title and abstract screening, of which 344 were excluded as not relevant. The remaining 55 reports were assessed for eligibility in full text; 45 were excluded with reasons (non-human or preclinical report, *n* = 14; conference abstract without sufficient denominator data, *n* = 10; non-English-language publication without available translation, *n* = 7; narrative review without original patient- or trial-level data, *n* = 14). A total of 10 data sources were included in the qualitative synthesis. Quantitative meta-analysis was not performed because of heterogeneity in study design, hemorrhage definitions, and reporting frameworks across included sources

Extracted variables included study design and enrollment period, patient demographics, tumor type, location, and molecular alterations, targeted agents and regimen, cumulative exposure or time on therapy, timing of hemorrhage relative to therapy initiation, hemorrhage phenotype (intratumoral [ITH] versus intracranial [ICH]), Common Terminology Criteria for Adverse Events (CTCAE) grade, concomitant or prior radiation therapy (including stereotactic radiosurgery), use of anticoagulant or antiplatelet agents, management, and clinical outcome including mortality. Given the heterogeneity of reporting, rarity of events, and lack of standardized CNS-specific bleeding definitions across trials, quantitative meta-analysis was not performed; results were synthesized narratively and in summary tables. Where denominators were unclear, we relied on the regulatory or primary trial publication as the preferred source. Title and abstract screening was performed independently by S.D. and A.C.; full-text review was conducted by S.D. with verification by A.C.; discrepancies were resolved by consensus with M.S.A. No automation tools were used. Data were extracted by S.D. and independently verified by A.C. No data were sought from original study investigators. Where available, hemorrhage events are expressed as incidence proportions (n/N); for the BRAFi + SRS meta-analysis, the reported odds ratio with 95% confidence interval is cited. A formal risk-of-bias assessment using a single validated instrument was not performed because the evidence base spanned heterogeneous designs for which no single appraisal tool is uniformly applicable. The resulting limitations, including susceptibility to selective reporting and ascertainment bias, are discussed narratively and should be weighed when interpreting the aggregate signal. The full Boolean search strings for each database are provided in the Supplementary Appendix to support reproducibility. The study selection process is summarized in a PRISMA flow diagram (Fig. 1).

## Results

### Study characteristics

The database search identified 580 records (PubMed/MEDLINE, *n* = 312; Embase, *n* = 268), supplemented by 25 records from FDA approval packages, prescribing information, and reference-list hand-searching. After removal of 206 duplicates, 399 records were screened at the title and abstract level, of which 344 were excluded. The remaining 55 reports were assessed in full text, and 45 were excluded with reasons (non-human or preclinical report, *n* = 14; conference abstract without sufficient denominator data, *n* = 10; non-English-language publication without available translation, *n* = 7; narrative review without original patient- or trial-level data, *n* = 14), yielding 10 data sources included in the synthesis (Fig. 1). The identified evidence base consisted of prospective clinical trials and their pooled safety updates, regulatory safety reviews and FDA prescribing information, post-marketing pharmacovigilance analyses, small observational cohorts, and individual case reports. No prospective study was specifically designed to evaluate CNS hemorrhage risk as a primary endpoint in MAPK-pathway–treated patients. Across all sources, ITH and ICH were infrequent but reproducibly reported, and when severe represented dose-limiting or fatal toxicities (Table 1).

**Table 1:** Reported intratumoral and intracranial hemorrhage associated with MAPK-pathway targeted therapy

Study / Dataset	Population	Agent(s)	*N*	Hemorrhage phenotype	CNS-specific hemorrhage, *n* (%)	Fatal hemorrhage, *n* (%)	Ref
BREAK-MB	Melanoma brain metastases	Dabrafenib	172	Intracranial	10 (6%)	0	[9]
COMBI-MB	Melanoma brain metastases	Dabrafenib + trametinib	125	Intracranial tumor hemorrhage	1 (0.8%)	1 (0.8%)	[10]
Pooled clinical-trial safety (dabrafenib-containing regimens)	Mixed (melanoma, NSCLC, thyroid, glioma)	Dabrafenib ± trametinib	1,087	Intracranial	7 (0.6%)	5 (0.5%)	[17]
FIREFLY-1	Pediatric BRAF-altered LGG (R/R)	Tovorafenib	137	Any-site hemorrhage 58 (42%); grade 3–4 in 7 (5%)	Serious intratumoral hemorrhage 8 (6%)	1 (0.7%) grade 5 intratumoral hemorrhage (after discontinuation, at progression)	[3]
Pooled tovorafenib safety population (FDA label)	Pediatric R/R LGG	Tovorafenib	140	Any-site hemorrhage 37%; intratumoral 9%	13 (9%) intratumoral	Not reported in label	[12]
TADPOLE (LGG)	First-line pediatric BRAF V600 LGG	Dabrafenib + trametinib vs. C/V	73	Epistaxis / bleeding (all sites) 25%	Rare isolated ICH reported	0	[4]
Pediatric HGG trial	R/R BRAF V600 pediatric HGG	Dabrafenib + trametinib	41	Intratumoral / intracranial	Rare; case-level only	0	[7]
SPRINT (NF1 PN)	Pediatric NF1 plexiform neurofibromas	Selumetinib	50	Bleeding — label warning (vitamin E effect)	Not reported	0	[8, 15]
BRAFi + SRS meta-analysis	Melanoma brain metastases	BRAF ± MEK inhibitor + SRS	976 pooled; ICH subset smaller	Intracranial hemorrhage	OR 3.16 (95% CI 1.43–6.96) vs. SRS alone	Variable	[18]
Lee et al. (case report)	Melanoma brain metastases	Dabrafenib + trametinib	1	Intracranial hemorrhage	1	0	[19]

Where available, denominators reflect the primary trial publication or FDA prescribing information. CNS hemorrhage figures include both intratumoral and non-intratumoral intracranial events unless otherwise noted; all-site bleeding figures (including epistaxis) are confined to the hemorrhage phenotype column and are not CNS events. FIREFLY-1 reporting aggregated all-site bleeding events; the 42% figure includes predominantly minor (grade 1–2) events such as epistaxis, approximately half of which were asymptomatic. The FIREFLY-1 safety population (*n* = 137) and the pooled FDA-label safety population (*N* = 140) overlap substantially and represent the same clinical program; the 42% any-site figure derives from the former and the 9% intratumoral figure from the latter, and the two should not be summed. Serious intratumoral hemorrhage counts for FIREFLY-1 are drawn from the updated safety report [20]. For the BRAFi + SRS meta-analysis, *n* = 976 across eight studies denotes the pooled sample for the survival and local-control analyses; the intracranial-hemorrhage comparison derives from a smaller subset of those studies. BMs, brain metastases; C/V, carboplatin/vincristine; HGG, high-grade glioma; ICH, intracranial hemorrhage; LGG, low-grade glioma; NF1, neurofibromatosis type 1; NSCLC, non–small cell lung cancer; OR, odds ratio; PN, plexiform neurofibroma; R/R, relapsed/refractory; SRS, stereotactic radiosurgery

### Dabrafenib and dabrafenib/trametinib in melanoma brain metastases

Dabrafenib-based regimens in melanoma brain metastases have produced the most mature adult CNS safety dataset. In the phase 2 BREAK-MB trial of dabrafenib monotherapy (*n* = 172), ICH was reported in 10 patients (6%); no hemorrhagic events were fatal and were attributed to underlying melanoma metastases rather than a direct drug effect [9]. In the phase 2 COMBI-MB trial of dabrafenib plus trametinib (*n* = 125), a single fatal intracranial tumor hemorrhage was reported (1 of 125; 0.8%); other serious bleeding events were uncommon [10]. In pooled clinical-trial safety data for dabrafenib-containing regimens across indications (*N* = 1,087), ICH occurred in 7 patients (0.6%), including 5 fatal events (0.5%) [17]. Five-year follow-up of COMBI-d and COMBI-v did not identify new late-emerging CNS hemorrhage signals with long-term dabrafenib/trametinib exposure [21]. Collectively, these data suggest that dabrafenib-based regimens in adult melanoma populations are associated with a low absolute rate of severe CNS bleeding, but that events are reproducibly observed and occasionally fatal.

*Lee et al.* reported the case of a 48-year-old man with metastatic melanoma of unknown primary origin. The patient had metastases involving the right clavicle, liver, adrenal gland, and a left frontotemporal brain lesion, and underwent gross total resection followed by adjuvant CyberKnife stereotactic irradiation. The disease progressed after two prior lines of therapy, and treatment with dabrafenib plus trametinib was subsequently initiated. Four months after starting therapy, the patient presented with a severe headache and experienced a seizure. Brain MRI demonstrated extensive edema in the left frontal lobe with associated midline shift. An emergency craniotomy was performed, during which an intracranial hemorrhage was identified intraoperatively. Histopathological examination of the surgical specimen revealed organizing hemorrhage and necrosis with surrounding gliosis, without evidence of viable tumor cells [19].

### MAPK-pathway therapy in primary CNS tumors and NF1

Reports in primary CNS tumors outside pLGG are limited. In a phase 2 trial of dabrafenib plus trametinib in BRAF V600–mutant pediatric high-grade glioma (*n* = 41), hemorrhagic events were not highlighted as a major toxicity, but the small cohort size limits precision [7]. In the TADPOLE first-line pLGG trial comparing dabrafenib plus trametinib with carboplatin/vincristine, epistaxis and other bleeding events (combined) were reported in 25% of patients receiving the targeted combination, with isolated ICH events described but not systematically quantified [4]. In the phase 1/2 pediatric trametinib program, bleeding events were infrequent and primarily cutaneous or mucosal [5]. For selumetinib in NF1-associated plexiform neurofibromas, the SPRINT trial did not identify excess clinically significant bleeding in the pediatric cohort [8], but the FDA label includes an increased risk of bleeding as a Warning and Precaution, attributed in part to the vitamin E (α-tocopheryl) excipient in the selumetinib capsule formulation, which may impair platelet function and potentiate antithrombotic effects [15].

In the multicenter phase 2 PBTC-029B trial of selumetinib in children with recurrent or progressive low-grade glioma (*N* = 130 across strata), one of two patients in stratum 6 discontinued treatment because of a grade 2 intratumoral hemorrhage accompanied by a grade 3 headache, both considered possibly related to the study drug. Stratum 6 comprised patients with non-NF1-associated recurrent or progressive pLGG whose molecular characterization could not be completed because of insufficient tumor tissue or assay failure, but who otherwise met all study eligibility criteria. No other clinically significant intratumoral or intracranial hemorrhages were reported as treatment-related toxicity; bleeding events were otherwise limited to minor mucocutaneous events [22]. The absence of a broader CNS hemorrhage signal in this cohort may reflect the lower intrinsic hemorrhagic propensity of the enrolled tumor types (predominantly pilocytic astrocytoma) relative to melanoma brain metastases or MAPK-altered pLGG subgroups with more aggressive histology. The TADPOLE trial as described by Bouffet et al. is the largest prospective pediatric study of first-line dabrafenib plus trametinib in BRAF V600–mutant pLGG and reported isolated ICH events in the targeted therapy arm, though systematic CNS-specific hemorrhage data were not a pre-specified endpoint [4]. No grade 3 or higher ITH or ICH was attributed to dabrafenib plus trametinib as a treatment-related event in that population.

### Tovorafenib in pediatric low-grade glioma

In the pediatric setting, tovorafenib has produced the most striking hemorrhage signal to date. In FIREFLY-1 (*n* = 137 across arms 1 and 2), hemorrhagic events of any type occurred in 58 patients (42%); grade 3–4 events were reported in 7 patients (5%) and a separate single grade 5 (fatal) tumor hemorrhage occurred (1 of 137; 0.7%), which is not included among the seven grade 3–4 events. The fatal event occurred in a patient with disseminated leptomeningeal mixed glial-neuronal tumor and developed 21 days after the final dose of tovorafenib, which had previously been discontinued because of disease progression [3, 20]. Among the four patients with serious treatment-related tumor hemorrhage in FIREFLY-1, two had a documented history of intratumoral hemorrhage before starting tovorafenib, and all four of these serious events resolved [3]. Together with the fatal event described above, which occurred after drug discontinuation at the time of tumor progression, these observations indicate that prior hemorrhage and the natural history of the tumor are important determinants of the uncommon symptomatic CNS hemorrhage observed with tovorafenib, and suggest that the likelihood of unexpected treatment-induced symptomatic hemorrhage is lower than the aggregate 42% any-site figure might imply. Regarding timing, the events across the reviewed literature did not occur uniformly on active, responding therapy. The fatal pediatric event occurred 21 days after drug discontinuation for tumor progression, when circulating drug levels would have been minimal and progressive disease itself is a plausible contributor. Among the remaining severe events, some arose during active treatment, typically within the early response window, while others occurred in the setting of progressive disease or in patients with pre-existing intratumoral hemorrhage. Most primary reports, however, did not specify time on therapy or disease status at the time of bleeding, so this distinction cannot be quantified across the pooled evidence base and represents a priority for prospective capture.

Because the trial grouped bleeding events across all anatomic sites, the 42% figure encompasses predominantly minor events (most commonly epistaxis); CNS tumor hemorrhage represented the clinically severe tail of this distribution. In the pooled safety population informing FDA labeling (*N* = 140), hemorrhagic events occurred in 37% of patients overall, with intratumoral hemorrhage in 9% and epistaxis in 26%. “Hemorrhage” is listed among the most common (≥ 30%) adverse reactions and is included as a labeled Warning and Precaution [12]. These observations informed FDA-label language requiring baseline assessment of bleeding risk, monitoring during therapy, and dose interruption or discontinuation following significant hemorrhagic events [12]. Three tovorafenib denominators recur in the literature and should be distinguished: the registrational efficacy population (*n* = 76) used for the RAPNO-LGG response analysis, the FIREFLY-1 safety population (*n* = 137), and the pooled safety population informing FDA labeling (*N* = 140). The 42% any-site figure derives from the *n* = 137 safety population, whereas the 9% intratumoral hemorrhage figure derives from the *N* = 140 pooled population; these cohorts substantially overlap and should not be summed.

### Interaction with CNS-directed radiation

A consistent secondary signal involves combination of MAPK-pathway inhibitors with CNS-directed irradiation. In a systematic review and meta-analysis evaluating BRAF inhibitors with stereotactic radiosurgery (SRS) versus SRS alone in melanoma brain metastases, combination therapy was associated with higher odds of ICH (odds ratio 3.16; 95% CI 1.43–6.96; *p* = 0.004), despite improved survival and local control [18]. The pooled cohort of eight studies (*n* = 976) reported in that review informed the survival and local-control analyses; the intracranial-hemorrhage comparison was derived from a smaller, clinically and methodologically heterogeneous subset of these studies, and the odds ratio should be interpreted with that caveat. Observational data similarly suggest increased rates of radiation necrosis and symptomatic hemorrhage when BRAF inhibitors are delivered in close temporal proximity to SRS [23]. These findings are confounded by the underlying high hemorrhagic propensity of melanoma brain metastases and by variable intervals between systemic therapy and irradiation, but they support caution in timing and sequencing.

### Pharmacovigilance and drug labeling

Regulatory labels and post-marketing data reinforce the hemorrhagic signal. Prescribing information for tovorafenib [12], selumetinib [15], and dabrafenib [17] each contain explicit bleeding-related warnings, with selumetinib additionally noting vitamin E content and interaction with antithrombotic agents. Spontaneous adverse event reporting databases capture rare, serious hemorrhagic events not fully reflected in controlled trials, although causal attribution in such reports is limited by confounding and incomplete denominators.

## Discussion

This systematic review identifies a consistent but heterogeneous signal of intratumoral and intracranial hemorrhage associated with MAPK-pathway targeted therapies across multiple tumor types and treatment settings. Although absolute incidence is low in most adult datasets, the presence of severe and occasionally fatal events in CNS tumor populations underscores the clinical relevance of this toxicity. The markedly higher aggregated bleeding rate reported with tovorafenib (37–42% any-grade in FIREFLY-1 and the pooled safety population) [3, 12] must be interpreted in the context of differences in reporting: tovorafenib trials captured minor bleeding events (particularly epistaxis) that were likely undercounted or grouped differently in older trials of dabrafenib-based regimens. The clinically severe fraction (grade 3–4) events in 5% and fatal tumor hemorrhage in < 1% is broadly similar in magnitude to the severe CNS bleeding signal in dabrafenib-exposed populations.

**Fig. 2:**
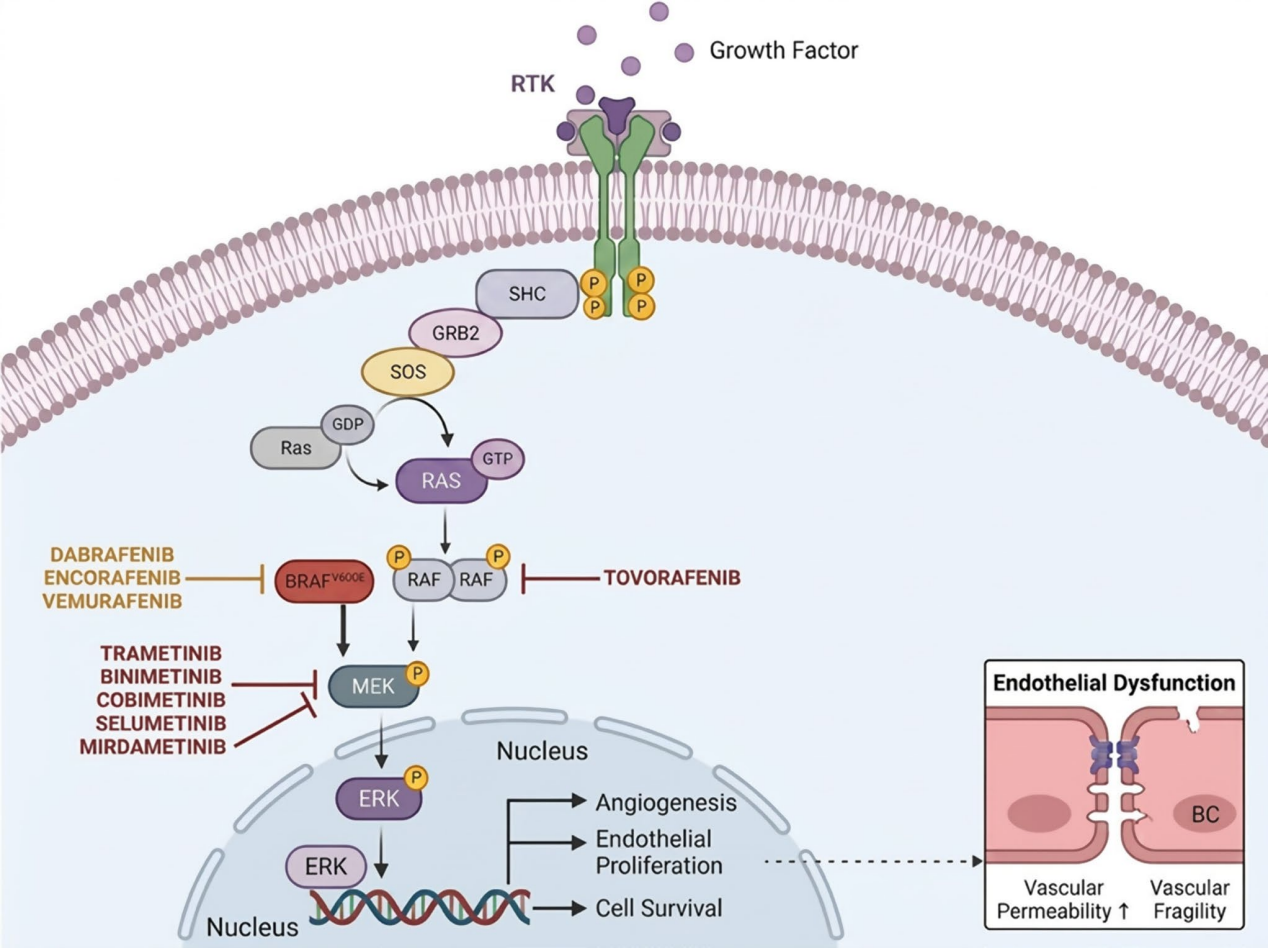
RAS–RAF–MEK–ERK signaling cascade and proposed mechanisms linking MAPK-pathway inhibition to endothelial dysfunction and CNS hemorrhage. Growth factor binding to a receptor tyrosine kinase (RTK) recruits the SHC–GRB2–SOS adaptor complex, activating RAS and driving sequential phosphorylation of RAF, MEK, and ERK. Nuclear ERK regulates transcription of genes governing angiogenesis, endothelial proliferation, and cell survival. Pharmacologic inhibition occurs at three nodes: type I BRAF V600E inhibitors (dabrafenib, encorafenib, vemurafenib); the type II RAF inhibitor tovorafenib, active against both BRAF fusions and V600 alterations; and MEK inhibitors (trametinib, binimetinib, cobimetinib, selumetinib, mirdametinib). Because physiologic VEGF receptor-2 signaling in endothelial cells converges on this same axis to maintain vascular integrity, pathway blockade compromises endothelial homeostasis, increasing vascular permeability and fragility. In parallel, MAPK inhibition downregulates tumor cell tissue factor expression and, through rapid cytoreduction, destabilizes disorganized tumor-associated vasculature, together providing a plausible mechanistic framework for the intratumoral and intracranial hemorrhage observed across BRAF, MEK, and type II RAF inhibitor exposure. BC, blood cell; ERK, extracellular signal-regulated kinase; GRB2, growth factor receptor–bound protein 2; MEK, mitogen-activated protein kinase kinase; RTK, receptor tyrosine kinase; SHC, Src homology 2 domain–containing transforming protein; SOS, son of sevenless; VEGF, vascular endothelial growth factor

Several nonexclusive biological mechanisms are plausible (Fig. 2). MAPK-pathway inhibition can induce rapid tumor cell death and necrosis, particularly in the early response phase, which may destabilize tumor-associated vasculature and precipitate intratumoral bleeding. This mechanism is consistent with early-treatment timing of many severe events and with the higher rates observed in highly vascular tumors such as melanoma brain metastases [14]. BRAF is known to play a fundamental role in the regulation of angiogenesis during embryonic development. In this context, the work by Wojnowski et al. identified BRAF as a critical signaling mediator in vascular system formation and provided the first genetic evidence supporting an essential role of RAF genes in the regulation of programmed cell death [24]. Beyond embryogenesis, the RAS–RAF–MEK–ERK signaling cascade also exerts a protective role within the vasculature through its interaction with vascular endothelial growth factor (VEGF). In endothelial cells, activation of VEGF receptor-2 stimulates phospholipase C, leading to downstream activation of the RAF–MEK–ERK pathway and promoting endothelial cell proliferation [25–27]. Furthermore, in vitro work by Scatena et al. has demonstrated that the BRAF inhibitor dabrafenib and the MEK inhibitor trametinib downregulate tissue factor (TF) expression in BRAF V600E–mutated melanoma cells, resulting in suppression of the coagulation cascade [28]. TF, a transmembrane glycoprotein, is the principal initiator of physiological blood coagulation and is frequently overexpressed in several malignant tumors, including melanoma, where its expression has been associated with a hypercoagulable state. Collectively, these findings suggest that inhibition of the RAF–MEK–ERK pathway may influence vascular homeostasis and hemostasis through both endothelial and coagulation-related mechanisms.

Additionally, prior or concurrent CNS radiation therapy injures endothelium and disrupts the blood-brain barrier, creating a permissive environment for hemorrhage; the odds ratio of 3.16 for ICH in BRAFi + SRS versus SRS alone supports this additive mechanism [18, 23]. Drug-specific factors may also contribute as the vitamin E (α-tocopheryl) excipient in the selumetinib capsule formulation potentiates bleeding risk through at least two convergent mechanisms. Vitamin E interferes with vitamin K metabolism, reducing activation of vitamin K-dependent coagulation factors and suppressing Factor IX synthesis [29, 30], and inhibits protein kinase C, impairing platelet activation and aggregation [31, 32]. Together these mechanisms provide a plausible basis for the labeled bleeding warning and support caution when selumetinib is co-administered with antithrombotic agents [15]. Type II RAF inhibitors such as tovorafenib may have distinct effects on tumor and normal endothelium that warrant further study. Finally, systemic modifiers including therapeutic anticoagulation, antiplatelet therapy, thrombocytopenia, and baseline coagulopathy almost certainly modulate risk, although these co-exposures are inconsistently reported. The absence of a significant CNS hemorrhage signal in selumetinib-treated pilocytic astrocytoma cohorts, in contrast to the higher rates in melanoma brain metastases and tovorafenib-treated pLGG, likely reflects the lower intrinsic hemorrhagic propensity of those tumor types rather than a drug-specific protective effect; this observation should be interpreted with caution given the absence of routine susceptibility-weighted MRI surveillance in most of these studies. Published case reports of ITH or ICH specifically attributable to MAPK-pathway inhibitors in primary CNS tumor populations outside the trials summarized above remain limited, representing a gap in the literature that prospective imaging-based studies could address. As a reference point, spontaneous hemorrhage is common in the tumor types under study even in the absence of targeted therapy: melanoma brain metastases carry a high intrinsic hemorrhagic propensity [14], and spontaneous intratumoral hemorrhage in pediatric low-grade glioma has been reported at rates of approximately 7–20% [20], overlapping the symptomatic hemorrhage rates observed on tovorafenib. Tumor molecular subtype is itself an important and under-recognized determinant of baseline hemorrhagic risk. FGFR1 alterations in particular have been associated with a high rate of spontaneous intracranial hemorrhage independent of targeted therapy [33, 34]. Notably, FIREFLY-1 enrolled BRAF-altered tumors and did not include patients with known FGFR mutations or fusions. The contribution of tumor genotype to the observed hemorrhage signal therefore cannot be fully disentangled from any direct drug effect, and reported incidence should not be generalized across molecular subgroups. Whether specific tumor locations confer differential hemorrhage risk could not be determined from the available data.

From a clinical standpoint (Table 2), several practical points emerge. Baseline assessment of hemorrhage risk including prior intratumoral bleeding, tumor vascularity, concomitant medications, and recent or planned CNS-directed irradiation should inform treatment selection and monitoring strategy. Baseline MRI with T2 susceptibility-weighted imaging can identify pre-existing microhemorrhage that may refine risk stratification. Because susceptibility-weighted imaging is already routine in surveillance protocols at most high-volume centers and incidentally detected microhemorrhages seldom alter the decision to continue targeted therapy, its principal value in this setting is baseline risk stratification rather than as a stand-alone trigger for intervention. The early treatment window (first ~ 4–8 weeks), during which tumor response and vascular remodeling are most pronounced, warrants heightened vigilance and a low threshold for interval imaging in response to new or worsening neurologic symptoms. When concurrent CNS-directed radiation therapy is necessary, careful sequencing with attention to temporal proximity of SRS and MAPK-inhibitor exposure may mitigate additive risk. Integration of CNS-specific hemorrhage endpoints into MAPK-pathway inhibitor trials (with standardized CTCAE grading, imaging confirmation, and separation of CNS from non-CNS bleeding) would substantially improve the ability to define true incidence and risk modifiers. Considered together, the risk modifiers in Table 2 span patient-related (therapeutic anticoagulation, antiplatelet use, thrombocytopenia), treatment-related (temporal proximity to SRS, combination therapy, the early-response window), drug-specific (the selumetinib vitamin E excipient, the tovorafenib labeled warning), tumor-related (vascularity, prior hemorrhage, molecular subtype), and clinical-context (confined intracranial compartment, pediatric age) domains; explicit attention to each domain at baseline and during early therapy provides a practical framework for individualized monitoring.

**Table 2:** Clinical risk modifiers and contributing factors for CNS hemorrhage with MAPK-pathway targeted therapy

Category	Factor	Mechanism / rationale	Clinical implication
Tumor-related	Highly vascular tumors (e.g., melanoma brain metastases)	Fragile, disorganized tumor vasculature with high baseline hemorrhage propensity	Higher baseline ICH risk; consider closer imaging surveillance
Tumor-related	Large tumor burden or pre-existing necrosis	Rapid cytoreduction and vascular destabilization upon MAPK inhibition	Highest-risk window in first weeks after initiation
Tumor-related	Prior intratumoral hemorrhage	Pre-existing vascular fragility and hemosiderin deposition	Assess baseline MRI with T2*/SWI; individualize risk–benefit
Treatment-related	MAPK inhibitor initiation / early response	Tumor shrinkage and vascular remodeling	Interval imaging at 4–8 weeks; clinical vigilance for symptoms
Treatment-related	Concurrent or recent stereotactic radiosurgery (SRS) or fractionated RT	Endothelial injury, radiation vasculopathy, disrupted blood–brain barrier	Avoid tight temporal pairing with SRS when feasible; OR for ICH ≈ 3.16 in melanoma BMs
Treatment-related	BRAF + MEK combination therapy	Enhanced response depth and possible faster necrosis	Close monitoring during first cycles
Patient-related	Therapeutic anticoagulation	Impaired hemostasis	Review indication; prefer agents with reversal options if needed
Patient-related	Antiplatelet therapy	Impaired primary hemostasis	Weigh bleeding vs. thrombotic risk; coordinate with treating team
Patient-related	Thrombocytopenia or coagulopathy	Reduced hemostatic reserve	Correct reversible factors prior to initiation
Drug-specific	Selumetinib (vitamin E excipient)	Possible platelet dysfunction from α-tocopheryl-containing formulation	Caution with concomitant anticoagulants/antiplatelets; labeled warning
Drug-specific	Tovorafenib	Hemorrhage is a labeled warning; 37% any-site, 9% intratumoral in the pooled (*N* = 140) safety population	Enhanced surveillance; dose interruption/modification per label
Drug-specific	Dabrafenib ± trametinib	Rare ICH; highest signal in melanoma BMs or when combined with SRS	Screen for co-exposures (RT, anticoagulants) prior to initiation
Clinical context	Confined intracranial compartment	Small volumes of blood → substantial neurologic impact	Lower threshold for neurosurgical consultation
Clinical context	Pediatric population	Longer treatment duration; developing vasculature; limited reversal options	Age-appropriate monitoring; family education on warning signs

BMs, brain metastases; ICH, intracranial hemorrhage; MRI, magnetic resonance imaging; RT, radiation therapy; SRS, stereotactic radiosurgery; SWI, susceptibility-weighted imaging

Across the broader evidence base, there is no support for preemptive dose reduction of BRAF inhibitors as a strategy to prevent bleeding events, nor any justification for withholding anticoagulation solely on the basis of initiating BRAF-targeted therapy. Consequently, decisions regarding anticoagulation should be individualized, carefully balancing the patient’s bleeding risk profile against the clinical indication for anticoagulation [25, 35]. This uncertainty is further reflected in the Delphi consensus [36], which considered both cutaneous and non-cutaneous adverse events. However, hemorrhagic toxicity was not specifically addressed within the non-cutaneous domain, highlighting a relevant gap in both the current evidence and expert agreement.

In clinical practice, management remains largely pragmatic. During acute hemorrhage, temporary interruption of BRAF inhibition is generally recommended, along with correction of reversible contributors such as concomitant use of NSAIDs, corticosteroids, or antithrombotic agents. For grade ≥ 3 bleeding events, treatment suspension should be strongly considered, together with evaluation and correction of any underlying coagulopathy in close collaboration with hematology specialists [35, 37]. In the absence of formal guidelines, these recommendations remain primarily grounded in expert opinion. With respect to laboratory monitoring, no evidence-based thresholds have been established; it is nonetheless reasonable to document a baseline platelet count and coagulation profile before initiation, correct clinically significant thrombocytopenia or coagulopathy, and reassess these parameters if bleeding symptoms arise, whereas routine scheduled coagulation testing in asymptomatic patients has not been shown to be necessary.

This review has several limitations. The available evidence is largely retrospective or derived from post hoc safety analyses of clinical trials that were not specifically designed to evaluate CNS hemorrhage as a primary outcome. Reporting bias is also likely, including potential underreporting of mild hemorrhagic events in earlier studies and overrepresentation of severe events in published case reports. In addition, key clinical variables such as the timing of bleeding onset after treatment initiation and the safety of treatment reintroduction following a hemorrhagic event were not consistently reported across the included studies. Where timing was described, events appeared to cluster in two settings, early during treatment, when tumor response and vascular remodeling are most pronounced, and, as in the fatal FIREFLY-1 case, at the time of disease progression after drug discontinuation, but systematic timing data, including the interval between CNS-directed radiation and hemorrhage onset and the number of pediatric patients irradiated before starting a MAPK inhibitor, were rarely available. Concomitant antiangiogenic therapy (e.g., bevacizumab) is a further plausible confounder of bleeding risk but was not reported in the included studies, so its contribution could not be assessed and should be captured prospectively. Hemorrhage definitions are inconsistent across trials, with some aggregating all bleeding events (including epistaxis), others reporting only grade ≥ 3, and few providing imaging-confirmed, CNS-specific denominators. These heterogeneities preclude quantitative meta-analysis and limit direct cross-agent comparison. Additionally, denominators in pharmacovigilance and case-level reports are often unavailable, so apparent signals in such datasets should be interpreted cautiously. A further inherent limitation is the absence of head-to-head comparisons of intracranial hemorrhage risk between conventional carboplatin/vincristine chemotherapy and RAF/MEK/ERK-targeted therapy; no included study directly compared CNS hemorrhage rates between these approaches, so their relative risk cannot be established. Relatedly, reported incidences are frequently based on non-equivalent endpoints. For example, < 1% symptomatic ICH with dabrafenib in adult melanoma versus 9% intratumoral hemorrhage with tovorafenib in pediatric pLGG that differ in population, hemorrhage definition, and ascertainment method; such figures should be compared only with these differences in view, and ideally restricted to matched endpoints (e.g., imaging-confirmed intratumoral hemorrhage or grade ≥ 3 events).

Despite these limitations, the consistency of reported severe and fatal events across multiple agents and independent datasets supports the existence of a real, albeit uncommon, CNS toxicity signal. As MAPK-pathway inhibitors continue to expand into earlier lines of therapy and broader CNS tumor populations including front-line pLGG [4], pediatric high-grade glioma [7], and NF1-associated tumors [8], prospective data collection with uniform CNS hemorrhage definitions is essential. Priority future directions include standardized CNS-specific bleeding endpoints in trials (distinguishing ITH, ICH, and non-CNS bleeding), prospective imaging surveillance with susceptibility-weighted MRI to identify subclinical microhemorrhage and correlate with clinically significant events, systematic capture of co-exposures (i.e., antithrombotics, radiation timing), and correlative studies of vascular integrity and molecular predictors of hemorrhage risk.

## Conclusion

Intratumoral and intracranial hemorrhage represent rare but clinically meaningful adverse events reported across MAPK-pathway targeted therapies, with severe events observed most consistently in CNS tumor populations. Reported incidence varies substantially by agent, population, and ascertainment method, and the true rate of clinically significant CNS hemorrhage likely lies between the narrow signal seen in adult melanoma trials and the broader aggregated bleeding rate reported in pediatric tovorafenib datasets. As MAPK-pathway inhibitors move into earlier lines and broader CNS indications, standardized hemorrhage definitions, prospective CNS-specific pharmacovigilance, and integrated risk stratification are needed to balance substantial therapeutic benefit with optimal patient safety.

## Supplementary Information

Below is the link to the electronic supplementary material.

Supplementary Material 1

Supplementary Material 2

## Data Availability

No datasets were generated or analysed during the current study.
