# Supplementary material for: Intratumoral and intracranial hemorrhage associated with MAPK-pathway targeted therapy: a systematic review and mechanistic synthesis
Source: J Neurooncol. 2026 Jul 15;178(3):103. doi: 10.1007/s11060-026-05714-0 (PMC13372930; doi:10.1007/s11060-026-05714-0)
Supplement: Supplementary file 1 — Supplementary Material 1 [file 11060_2026_5714_MOESM1_ESM.docx]

**PRISMA 2020 Checklist**

*Intratumoral and Intracranial Hemorrhage Associated with MAPK-Pathway Targeted Therapy: A Systematic Review and Mechanistic Synthesis*

| **Item #** | **Topic** | **Checklist item** | **Location / status** |
| --- | --- | --- | --- |
| **TITLE** | | | |
| 1 | Title | Identify the report as a systematic review. | **Title page — identifies the report as a systematic review.** |
| **ABSTRACT** | | | |
| 2 | Abstract | See the PRISMA 2020 for Abstracts checklist. | **Present. Structured abstract (Background, Methods, Results, Conclusions)** |
| **INTRODUCTION** | | | |
| 3 | Rationale | Describe the rationale for the review in the context of existing knowledge. | **Introduction — rationale established across MAPK-pathway agents and CNS populations.** |
| 4 | Objectives | Provide an explicit statement of the objective(s) or question(s) the review addresses. | **Introduction, final paragraph — explicit aims stated.** |
| **METHODS** | | | |
| 5 | Eligibility criteria | Specify the inclusion and exclusion criteria for the review and how studies were grouped for the syntheses. | **Methods, 'Eligibility criteria' — inclusion/exclusion specified.** |
| 6 | Information sources | Specify all databases, registers, websites, organisations, reference lists and other sources searched or consulted, with the date when each was last searched. | **Present. Methods, ‘Search strategy’ — PubMed/MEDLINE, Embase, FDA approval packages and prescribing information, reference-list hand-searching. Last searched May 29, 2026.** |
| 7 | Search strategy | Present the full search strategies for all databases, registers and websites, including any filters and limits used. | **Partial. Search terms summarized in Methods text. Full, line-by-line search strings per database not included in the manuscript** |
| 8 | Selection process | Specify the methods used to decide whether a study met the inclusion criteria, including how many reviewers screened each record, whether they worked independently, and any automation tools used. | **Present. Title and abstract screening was performed independently by S.D. and A.C.; full-text review was conducted by S.D. with verification by A.C.; discrepancies were resolved by consensus with M.S.A. No automation tools were used. Stated in the ‘Data extraction and synthesis’ paragraph.** |
| 9 | Data collection process | Specify the methods used to collect data from reports, including how many reviewers, whether independent, and any processes for obtaining or confirming data from investigators. | **Present. Extracted variables are defined. Data were extracted by S.D. and independently verified by A.C.; discrepancies were resolved by M.S.A. No data were sought from original study investigators. Stated in the ‘Data extraction and synthesis’ paragraph (Draft 12).** |
| 10a | Data items (outcomes) | List and define all outcomes for which data were sought; specify whether all results compatible with each were sought. | **Hemorrhage phenotype (ITH vs ICH), CTCAE grade, fatality defined in 'Data extraction'.** |
| 10b | Data items (other) | List and define all other variables for which data were sought (e.g., participant and intervention characteristics, funding). | **Agent/regimen, tumor type, denominator, radiotherapy and antithrombotic co-exposure defined.** |
| 11 | Study risk of bias | Specify the methods used to assess risk of bias, including details of the tool(s) used, how many reviewers, and whether independent. | **Present. Formal risk-of-bias assessment was not performed; study limitations are discussed narratively. Stated explicitly in the ‘Data extraction and synthesis’ paragraph). The Discussion limitations further acknowledge the retrospective, post-hoc nature of most included sources. No formal tool (e.g., ROBINS-I) was applied, consistent with the nature of the included sources (regulatory-grade trial safety data, prospective trials, one published case report).** |
| 12 | Effect measures | Specify for each outcome the effect measure(s) (e.g., risk ratio, mean difference) used in the synthesis or presentation of results. | **Present. Effect measures stated explicitly in the ‘Data extraction and synthesis’ paragraph (Draft 12): hemorrhage events expressed as incidence proportions (n/N); for the BRAFi+SRS meta-analysis, the reported odds ratio with 95% confidence interval is cited.** |
| 13a | Synthesis (eligibility) | Describe the processes used to decide which studies were eligible for each synthesis. | **Methods, 'Data extraction and synthesis' — narrative grouping by agent and population.** |
| 13b | Synthesis (preparation) | Describe any methods required to prepare the data for presentation or synthesis. | **Reliance on regulatory/primary publication where denominators unclear, stated.** |
| 13c | Synthesis (tabulation) | Describe any methods used to tabulate or visually display results of individual studies and syntheses. | **Summary tables (Tables 1–2) described; narrative synthesis.** |
| 13d | Synthesis (methods) | Describe any methods used to synthesize results and provide a rationale; if meta-analysis, describe model and software. | **Narrative synthesis; meta-analysis not performed, with rationale (heterogeneity, rarity, non-standard definitions) stated.** |
| 13e | Synthesis (heterogeneity) | Describe any methods used to explore possible causes of heterogeneity among study results. | **Sources of heterogeneity discussed narratively in Discussion; no quantitative exploration (none feasible).** |
| 13f | Synthesis (sensitivity) | Describe any sensitivity analyses conducted to assess robustness of the synthesized results. | **Not applicable — stated. Narrative synthesis only; no sensitivity analyses conducted or feasible. No pooled estimates generated (except the externally published BRAFi+SRS meta-analysis cited as ref 5).** |
| 14 | Reporting bias assessment | Describe any methods used to assess risk of bias due to missing results in a synthesis. | **Present. Reporting bias addressed qualitatively in Discussion limitations (underreporting of mild events in earlier trials; overrepresentation of severe events in case reports). Formal funnel plot or Egger test not applicable to narrative synthesis; explicitly stated as not performed.** |
| 15 | Certainty assessment | Describe any methods used to assess certainty (or confidence) in the body of evidence for an outcome. | **Stated. No formal GRADE certainty assessment performed, consistent with the narrative synthesis design and the heterogeneous, largely safety-reporting evidence base. This limitation is acknowledged in the Discussion. A GRADE assessment would require outcome-level pooled estimates, which are not available for this review.** |
| **RESULTS** | | | |
| 16a | Study selection | Describe the results of the search and selection process, ideally using a flow diagram, from records identified to studies included. | **Results, 'Study characteristics' (reconciliation sentence added) + PRISMA flow diagram (Figure 1).** |
| 16b | Study selection (exclusions) | Cite studies that might appear to meet the inclusion criteria but were excluded, and explain why. | **Exclusion reasons summarized in flow diagram.** |
| 17 | Study characteristics | Cite each included study and present its characteristics. | **Results subsections + Table 1 present per-study characteristics.** |
| 18 | Risk of bias in studies | Present assessments of risk of bias for each included study. | **Not presented (no formal RoB assessment — see item 11).** |
| 19 | Results of individual studies | For all outcomes, present for each study summary statistics and effect estimates, ideally with structured tables or plots. | **Per-study incidence and severity reported in text and Table 1.** |
| 20a | Results of syntheses | For each synthesis, briefly summarise the characteristics and risk of bias among contributing studies. | **Narrative summaries by agent/population in Results.** |
| 20b | Results of syntheses | Present results of all statistical syntheses; if meta-analysis, present summary estimate and precision. | **Narrative synthesis; the one pooled estimate cited (BRAFi+SRS OR 3.16) reported with 95% CI.** |
| 20c | Results of syntheses | Present results of all investigations of possible causes of heterogeneity. | **Discussed narratively (Discussion).** |
| 20d | Results of syntheses | Present results of all sensitivity analyses. | **Not applicable (narrative synthesis).** |
| 21 | Reporting biases | Present assessments of risk of bias due to missing results for each synthesis. | **Addressed qualitatively in Discussion limitations.** |
| 22 | Certainty of evidence | Present assessments of certainty (or confidence) in the body of evidence for each outcome. | **Not formally assessed (see item 15).** |
| **DISCUSSION** | | | |
| 23a | Discussion | Provide a general interpretation of the results in the context of other evidence. | **Discussion — interpretation in context, including mechanistic synthesis.** |
| 23b | Discussion | Discuss any limitations of the evidence included in the review. | **Discussion — evidence limitations (retrospective, inconsistent definitions, denominators).** |
| 23c | Discussion | Discuss any limitations of the review processes used. | **Present. Discussion limitations state: (1) evidence is retrospective or post-hoc; (2) no formal risk-of-bias tool applied; (3) screening and extraction performed by two reviewers with adjudication; (4) heterogeneous definitions preclude meta-analysis; (5) denominators unavailable in pharmacovigilance reports.** |
| 23d | Discussion | Discuss implications of the results for practice, policy and future research. | **Discussion — clinical implications and prioritized future directions.** |
| **OTHER INFORMATION** | | | |
| 24a | Registration | Provide registration information for the review, including register name and registration number, or state that the review was not registered. | **Present. Registration statement added to manuscript end matter, no registration.** |
| 24b | Protocol | Indicate where the review protocol can be accessed, or state that a protocol was not prepared. | **Present. Protocol statement added to manuscript end matter** |
| 24c | Amendments | Describe and explain any amendments to information provided at registration or in the protocol. | **Present. Amendments statement added to manuscript.** |
| 25 | Support | Describe sources of financial or non-financial support and the role of funders. | **Present. Funding section present in manuscript end matter: “No funding was received for this work.”** |
| 26 | Competing interests | Declare any competing interests of review authors. | **Present. Conflict of Interest section present in manuscript end matter.** |
| 27 | Availability of data, code and other materials | Report which of the following are publicly available and where: data, analytic code, other materials used. | **Present. Data Availability statement present in manuscript end matter: all data derived from published studies, regulatory documents, and prescribing information cited in the reference list; no original patient-level data were generated or analyzed; all source documents are publicly available.** |

***Note.*** *Location and status entries reflect the state of Draft 12 (final submission version, May 31, 2026). All items are fully addressed except item 7: full database search strings should be provided as a supplementary appendix. All other previously partial or absent items (8, 9, 11, 12, 24a, 24b, 24c) have been resolved in Draft 12. The PRISMA flow-diagram counts (580 records identified from databases, 25 additional records, 206 duplicates removed, 399 records screened, 344 excluded at title/abstract, 55 full-text assessed, 45 excluded with reasons [n=14 preclinical, n=10 conference abstract, n=7 non-English, n=14 narrative review], 10 data sources included) reflect the updated search through May 29, 2026 and are consistent with the current manuscript text (Results, ‘Study characteristics’) and Figure 1.*

**Reference:** Page MJ, McKenzie JE, Bossuyt PM, et al. The PRISMA 2020 statement: an updated guideline for reporting systematic reviews. BMJ. 2021;372:n71.
