# Supplementary material for: Intratumoral and intracranial hemorrhage associated with MAPK-pathway targeted therapy: a systematic review and mechanistic synthesis
Source: J Neurooncol. 2026 Jul 15;178(3):103. doi: 10.1007/s11060-026-05714-0 (PMC13372930; doi:10.1007/s11060-026-05714-0)
Supplement: Supplementary file 2 — Supplementary Material 2 [file 11060_2026_5714_MOESM2_ESM.docx]

**Supplementary Appendix**

*Intratumoral and Intracranial Hemorrhage Associated with MAPK-Pathway Targeted Therapy: A Systematic Review and Mechanistic Synthesis*

Damodharan S, Calderon A, Abdelbaki MS

# **Supplementary Table S1. Full line-by-line search strategies**

Searches were executed from database inception through May 29, 2026. No language, date, or study-design limits were applied at the search stage; exclusions were applied at screening. Results were exported to a reference manager and de-duplicated prior to title/abstract screening.

## **S1.1 PubMed/MEDLINE (searched May 29, 2026; 312 records)**

#1 "intratumoral hemorrhage"[tiab] OR "intratumoural haemorrhage"[tiab] OR

"intracranial hemorrhage"[tiab] OR "intracranial haemorrhage"[tiab] OR

"tumor hemorrhage"[tiab] OR "tumour haemorrhage"[tiab] OR

"cerebral hemorrhage"[tiab] OR "bleeding"[tiab] OR

"Intracranial Hemorrhages"[Mesh]

#2 "BRAF inhibitor"[tiab] OR "MEK inhibitor"[tiab] OR "RAF inhibitor"[tiab] OR

"type II RAF inhibitor"[tiab] OR "MAPK pathway inhibitor"[tiab] OR

dabrafenib[tiab] OR vemurafenib[tiab] OR encorafenib[tiab] OR

tovorafenib[tiab] OR trametinib[tiab] OR cobimetinib[tiab] OR

binimetinib[tiab] OR selumetinib[tiab] OR mirdametinib[tiab] OR

"Proto-Oncogene Proteins B-raf/antagonists and inhibitors"[Mesh]

#3 #1 AND #2

## **S1.2 Embase (Ovid interface; searched May 29, 2026; 268 records)**

1 (intratumoral adj2 h?emorrhage).ti,ab.

2 (intracranial adj2 h?emorrhage).ti,ab.

3 (tumo?r adj2 h?emorrhage).ti,ab.

4 bleeding.ti,ab.

5 exp brain hemorrhage/

6 1 or 2 or 3 or 4 or 5

7 (BRAF adj2 inhibitor*).ti,ab.

8 (MEK adj2 inhibitor*).ti,ab.

9 (RAF adj2 inhibitor*).ti,ab.

10 (dabrafenib or vemurafenib or encorafenib or tovorafenib or trametinib

or cobimetinib or binimetinib or selumetinib or mirdametinib).ti,ab,rn.

11 7 or 8 or 9 or 10

12 6 and 11

## **S1.3 Supplementary sources (25 records)**

US Food and Drug Administration approval packages and current prescribing information for tovorafenib (OJEMDA), selumetinib (KOSELUGO), and dabrafenib (TAFINLAR) were reviewed directly. Reference lists of all included articles and of relevant narrative reviews were hand-searched for additional eligible reports.

# **Supplementary Note S2. Rationale for omission of a formal risk-of-bias assessment**

The included evidence base spanned prospective phase 1–2 trials, regulatory safety summaries, post-marketing pharmacovigilance analyses, small observational cohorts, and individual case reports. No single validated appraisal instrument (e.g., RoB 2, ROBINS-I, or the JBI case-report checklist) is applicable across this range of designs, and applying different instruments to different subsets would not yield a comparable summary judgment. Because no quantitative pooling was undertaken, a formal risk-of-bias score would not have altered any weighted estimate.
